# Pathogen Inactivation of Cellular Blood Products—An Additional Safety Layer in Transfusion Medicine

**DOI:** 10.3389/fmed.2017.00219

**Published:** 2017-12-04

**Authors:** Axel Seltsam

**Affiliations:** ^1^German Red Cross Blood Service NSTOB, Institute Springe, Springe, Germany

**Keywords:** transfusion, platelets, pathogen inactivation, ultraviolet light, red blood cells

## Abstract

In line with current microbial risk reduction efforts, pathogen inactivation (PI) technologies for blood components promise to reduce the residual risk of known and emerging infectious agents. The implementation of PI of labile blood components is slowly but steadily increasing. This review discusses the relevance of PI for the field of transfusion medicine and describes the available and emerging PI technologies that can be used to treat cellular blood products such as platelet and red blood cell units. In collaboration with the French medical device manufacturer Macopharma, the German Red Cross Blood Services developed a new UVC light-based PI method for platelet units, which is currently being investigated in clinical trials.

## Introduction

From the late 1970s to the mid-1980s, contaminated hemophilia blood products were a serious public health problem, resulting in the infection of large numbers of hemophiliacs with the human immunodeficiency virus (HIV). If safety measures had been implemented in a timely and consistent manner after identification of the acquired immune deficiency syndrome (AIDS) epidemic in 1981 and isolation of the HIV in 1983, the transmission of HIV infection by these blood products could have been prevented in most cases. This contaminated blood scandal made the community aware that new pathogens may emerge and threaten blood safety at any time. However, there was a significant delay in the introduction of HIV detection systems in some countries and in some cases, the detection tests that were implemented proved to be unreliable. In addition, the plasma products used for therapy were not even treated by heat inactivation—a pathogen inactivation (PI) method that was readily available and approved at that time. Consequently, blood and blood components became subject to drug law in some countries (1, 2).

Increasingly stringent donor eligibility criteria and more sensitive virus detection methods have reduced the risk of transfusion-transmitted infection (TTI) by blood products significantly, but a residual risk of TTI with viruses, bacteria, protozoa, and prions remains. False-negative test results due to test failures, very low-pathogen concentrations in the peripheral blood or escaped mutants can result in TTI in spite of negative screening tests (e.g., for *Treponema pallidum*, hepatitis B, hepatitis C, and HIV). In addition, transfusion recipients may be infected by pathogens not targeted in regular blood donor screening programs (e.g., hepatitis A and bacteria). Transfusion safety is particularly susceptible to pathogens that enter regions in which they are not yet endemic. The fact that viruses that are usually endemic in tropical regions have recently caused outbreaks in Western countries demonstrates that these pathogens can arise and threaten transfusion safety at any time (3, 4).

Blood safety is still mainly based on the reactive principle of introducing new test systems or new donor election criteria after a threat to transfusion recipients has been identified. In other words, infections by contaminated blood products must first occur before appropriate counter-measures are established. At the beginning of the last decade, a number of cases of West Nile virus occurred in the USA through the transmission of blood components before the first detection system for donor testing was implemented (3). The recent Zika virus outbreak on the American continent has heightened concerns over this reactive approach to blood supply safety (5, 6).

During an international consensus conference, transfusion experts and other stakeholders in the field of transfusion medicine recommended a change from the hitherto reactive strategy toward a proactive, preventive approach to blood safety (7). Recently, developed and approved PI technologies for cellular blood products, such as red blood cell (RBC) and platelet units, are considered key measures for closing or at least reducing the safety gap caused by emerging pathogens. While virus reduction procedures are an integral part of the process of manufacturing plasma derivatives from plasma pools, and although the methylene blue system has been used for PI of single donor plasma units for nearly two decades (8), a new generation of PI methods for platelet units have recently become available (9, 10). PI technologies for the treatment of RBC units are still in development and have not received market authorization yet.

## Technologies

The use of PI technologies for blood products has a number of advantages. Because they inactivate most clinically relevant viruses, bacteria, and protozoa, they can help to eliminate the residual risk of infection during the “window period” when transfusion-relevant pathogens (e.g., HIV) cannot be detected by donor screening tests. Their broad activity against pathogens also helps to reduce the risk of recognizable infectious agents (e.g., bacteria), which still cannot be prevented completely. In contrast to screening tests for transfusion-borne pathogens, PI proactively protects against emerging infectious agents entering the blood supply in a given community.

All PI methods used to treat cellular blood products work by impairing the target pathogen’s ability to replicate. When used alone or in combination, ultraviolet (UV) light and alkylating agents cause irreversible damage to the nucleic acids of pathogens. Therefore, they effectively eliminate classical pathogens such as viruses, bacteria, fungi, and protozoa, but are ineffective against prions. The latter protein-based pathogenic agents can cause sporadic and variant Creutzfeldt–Jakob disease in humans.

The following PI technologies for cellular products are currently available or in the pipeline.

### INTERCEPT Blood System for Platelets and Plasma

The INTERCEPT Blood System for platelets and plasma is manufactured by Cerus Corporation (Concord, CA, USA). The mechanism of action of this PI technology is based on the properties of amotosalen HCl (S-59), a photoactive compound which penetrates cellular and nuclear membranes and binds to the double-stranded regions of DNA and RNA. When activated by low-energy UVA light (320–400 nm), amatosalen cross-links nucleic acids and thus irreversibly blocks the replication of DNA and RNA (11). After illumination, residual amotosalen and its photoproducts must be removed during an incubation step lasting up to 16 h. The amatosalen/UVA procedure is not suitable for RBCs because of UVA light absorption by hemoglobin.

### MIRASOL PRT System for Platelets and Plasma

The MIRASOL system was developed by TerumoBCT (Lakewood, CO, USA). This photodynamic procedure employs riboflavin (vitamin B2) and broad spectrum UV light (mainly UVA und UVB, 285–365 nm). On exposure to UVA and UVB light, riboflavin associates with nucleic acids and mediates oxygen-independent electron transfer, causing irreversible damage to the nucleic acids (12). Because naturally occurring vitamin B2 and its photodegradation products are non-toxic and non-mutagenic, they do not need to be removed prior to transfusion. In addition to plasma and platelets, protocols for extension of the MIRASOL system to whole blood are now in development.

### THERAFLEX System for Platelets

**Figure 1:**
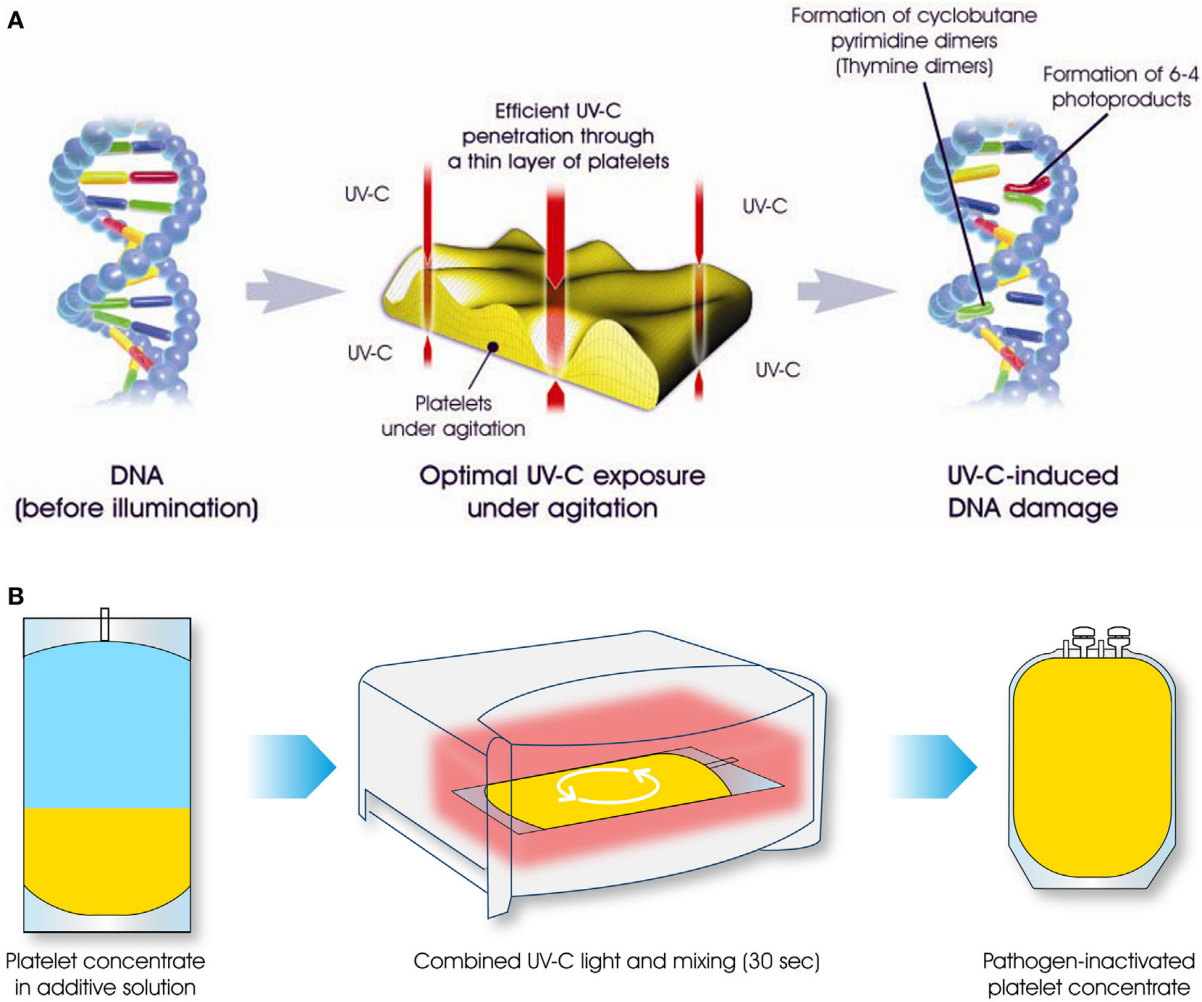
The THERAFLEX ultraviolet (UV)-Platelets pathogen inactivation system uses UVC light to induce irreversible damage to the nucleic acids of viruses, bacteria, fungi, protozoa, and leukocytes. Intense agitation of the platelet bag during UVC illumination results in efficient mixing, ensuring the uniform treatment of all blood compartments **(A)**. For the illumination step of this simple and rapid procedure, platelet units are placed in the irradiation device for a period of less than 1 min **(B)**. Afterward, the pathogen-reduced platelet product can be used for transfusion.

THERAFLEX UV-Platelets is a novel UVC-based PI technology that works without photoactive substances. It is the product of a joint venture between Macopharma (Mouvaux, France) and the German Red Cross Blood Service NSTOB in Springe, Germany. Shortwave UVC light (254 nm) directly interacts with nucleic acids to form pyrimidine dimers that block the elongation of nucleic acid transcripts (13). UVC irradiation mainly affects the nucleic acids of pathogens and leukocytes and does not impair plasma and platelet quality. As no photoactive substances are involved, UVC treatment is just as simple but faster (takes less than 1 min) than gamma irradiation, and can easily be integrated into the manufacturing processes at blood banks (Figure 1). The THERAFLEX system was originally developed for platelets but is also suitable for plasma and RBC units.

### S-303 PI System for RBCs

The S-303 PI system (INTERCEPT RBC system, Cerus Corporation, Concord, CA, USA) was specifically developed for RBC units. S-303 is a modular compound that prevents nucleic acid replication by targeting and cross-linking nucleic acids. Once added to the RBC unit, this amphipathic compound rapidly passes through cell and viral envelope membranes and intercalates into the helical regions of nucleic acids. S-300, the non-reactive byproduct of this reaction, is subsequently removed by incubation and centrifugation, which can take up to 20 h (14). In contrast to the other PI technologies described here, the S-303 system does not require UV light. However, glutathione (GSH), a naturally occurring antioxidant, must be used to prevent non-specific reactions between S-303 and other nucleophiles present in the RBC unit. These may include small molecules, such as phosphate and water, and macromolecules, such as proteins.

The INTERCEPT and MIRASOL systems for platelets and plasma have already been approved in the USA and some European and Asian countries, while both the THERAFLEX system and the S-303 system are still in clinical development. The UVC-based THERAFLEX system is expected to receive marketing authorization within the next few years (Table 1).

**Table 1:** Pathogen inactivation technologies.

	Technology			
	INTERCEPT blood system	MIRASOL PRT system	THERAFLEX UV-Platelets	S-303 system
Mechanism of action	UVA plus amotosalen (alkylating agent)	UV plus riboflavin (vitamin B2 = photosensitizer)	UVC alone	Alkylating agent
Blood products	Plasma and platelets	Plasma and platelets (in development for whole blood)	Plasma and platelets (in development for RBCs)	RBCs
Status	Approved in some countries	Approved in some countries	In clinical development	In clinical development

*UV, ultraviolet light; UVA, wavelength A; UVC, wavelength C; RBC, red blood cell*.

## Clinical Studies

### Platelets

Clinical studies show that platelets retain their hemostatic efficacy after PI treatment. Following prophylactic transfusion, there was no difference in the ability of pathogen-reduced and untreated platelet units to prevent severe bleeding (15). However, almost all clinical trials demonstrated that post-transfusion survival and recovery rates were consistently lower in patients receiving platelets treated with PI technology than in those transfused with untreated platelets (16–19). Accordingly, the transfusion of pathogen-reduced platelets resulted in lower platelet count increments (CIs), lower corrected count increments, shorter intervals between platelet transfusions, and a higher number of platelet transfusions per patient. However, observational studies showed no evidence of increased product consumption rates when pathogen-reduced platelet units were used in a routine setting (20).

Interestingly, the rate of acute transfusion reactions may be lower after the transfusion of pathogen-reduced versus untreated platelets. However, there have been concerns over acute respiratory distress associated with amatosalen/UVA-treated platelets (15). While the results of animal studies suggest that UV light-treated platelets mediated a higher risk of pulmonary toxicity (21), an analysis of clinical data by an expert panel does not confirm significant differences in the rates of acute lung disorders between PI-treated and untreated platelets (22). The results of ongoing large-scale phase III and hemovigilance studies will help to further clarify open questions with respect to therapeutic efficacy and potential side effects of pathogen-reduced platelets (23).

### Red Blood Cells

The S-303 system, which is in clinical development, is the only PI technology available for RBCs. Current studies are investigating the second-generation S-303 PI process. The first-generation S-303 procedure only marginally affected RBC quality and function, but after reports of immunization against pathogen-inactivated RBCs in transfused patients emerged, a new generation of the S-303 system had to be developed. In the second-generation S-303 system, the quencher concentration of GSH was increased from 2 to 20 mmol/l in order to decrease the affinity of S-303 for proteins and thus to avoid the formation of neoantigens on the surface of erythrocytes (24). However, recent studies show that immunization against S-303-coated RBCs still occurs after modification of the S-303 system (25). In particular, the fact that antibodies against S-303-treated cells were also detected in healthy blood donors who had never been transfused with pathogen-reduced RBCs suggests that some individuals may be immunized by S-303-like substances in the environment (e.g., food or air) or may have naturally occurring antibodies against epitopes on the S-303 molecule. These data clearly show that the use of chemical agents for PI of cellular products increases the risk of immune responses against blood components in transfusion recipients. Various phase III clinical trials to test the second-generation S-303 PI system for RBCs in acute and chronic anemia patients are currently ongoing or planned.

## Implementation in Routine Use

The INTERCEPT and MIRASOL PI systems for platelets and plasma are used in some parts of Asia, Europe, and the USA. In Europe, the willingness to use pathogen-reduced platelet units varies between countries and regions. PI technologies are implemented nationwide in some countries (e.g., Switzerland and Belgium), but only regionally in others (e.g., Poland). Evaluation of PI technologies for platelets is under way at some blood centers in Germany. In 2011, the Swiss national authority (Swissmedic) ordered the nationwide implementation of PI of platelet units. This measure was mainly aimed at preventing or at least minimizing the risk of fatal transfusion reactions caused by bacterially contaminated platelet units. Analysis of Swiss hemovigilance data revealed that without PI, one fatal case of transfusion-transmitted sepsis by contaminated platelet units would occur in Switzerland every 2 years. The US Food and Drug Administration (FDA) recently recommended the use of approved PI technologies as an alternative to bacterial detection methods in order to adequately control the risk of bacterial contamination of platelets (26, 27).

The preventive potential of PI of cellular blood components first became apparent during a chikungunya virus epidemic on the French island of La Reunion in the Indian Ocean in 2006 (28). Because more than 30% of the inhabitants were infected, local blood donation was suspended to prevent TTI. To sustain the availability of platelet components, the French national blood service (Etablissement Français du Sang) implemented universal PI of platelet components on the island. The success of this measure demonstrated that PI can effectively support the availability of safe labile blood components during an epidemic.

The West Nile virus epidemic in the USA was the first example of a large-scale arboviral threat to the blood supply of a Western country that required an urgent response across government agencies and non-governmental organizations. The dramatic spread of Zika virus in the Americas since 2015 has generated a sense of public health urgency akin to AIDS, along with immediate concerns over blood safety. In areas of active transmission, “FDA guidance recommends that blood be outsourced from unaffected areas, unless there are measures to screen donations using a laboratory test, or unless the blood components are subjected to PI technology” with an approved method (29). The INTERCEPT system was approved by the FDA in 2014 and has already been implemented at a number of US blood centers.

## Outlook

Despite the increasing and profound safety and efficacy record of pathogen-reduced blood cellular products, there are still concerns that impede the introduction of PI technology in hemotherapy. The INTERCEPT protocol includes incubation and adsorption steps that result in a significant loss of platelets (up to 15%) during preparation and PI treatment. However, this loss could be offset by performing PI with higher platelet counts in the starting products. The platelet yields could be increased by using more buffy coats (e.g., five instead of four) to manufacture pooled platelets, or by collecting higher numbers of platelets during the apheresis process. Moreover, this measure could compensate for reduced platelet CIs in transfusion recipients and thus lower the possible need for increased platelet unit utilization.

All PI technologies mentioned in this review exhibit gaps in their PI efficacy. The amatosalen/UVA-based system (INTERCEPT) is ineffective for non-enveloped viruses such as hepatitis A, hepatitis E, and parvovirus B19 (30). The riboflavin/UV-based system (MIRASOL) has only weakly effects against bacteria and some viruses (31). The UVC light-based system (THERAFLEX) is highly effective against bacteria and most transfusion-relevant viruses, but only moderately effective against HIV (32). However, when highly sensitive screening tests for HIV are performed, UVC-based PI could further reduce the risk of virus transmission during the “window period” in which the pre-nucleic acid testing can be negative and in patients with occult infections. Despite these weaknesses, PI systems generally have the potential to significantly add an additional layer of safety to blood transfusion.

Major concerns surrounding the implementation of PI have to do with its impact on the integrity of blood components and the toxicity of the chemicals used in these systems. In particular, acute and chronic toxicities may be caused by PI technologies that use active chemicals. Although only small quantities of photochemical compounds are used in PI technologies and they appear to provide sufficient safety margins, it cannot be excluded that alkylating agents such as amatosalen may be carcinogenic in the long term in a subset of transfused patients. A major advantage of the THERAFLEX system is that it works without photoactive substances, thus eliminating the risk of photoreagent-related adverse events (10, 13).

According to various stakeholders in the field of transfusion medicine, it is crucial to inactivate pathogens in all blood components in order to increase the safety margin of the entire blood supply. As long as PI is not routinely implemented in the production of RBC units (the most commonly used blood components), PI cannot achieve its full potential to enhance blood safety. Experts and health authorities are increasingly recommending the implementation of PI systems for platelets and plasma as an important step toward improving blood safety. A Canadian risk-benefit analysis suggests that if a new pathogen entered the blood supply, the use of pathogen-reduced plasma and platelets would reduce the risk of TTI by 40% (33).

The additional costs of PI implementation may be responsible for the hesitant acceptance of this technology by hospitals and funding agencies. Although based on assumptions and simplifications, the available cost-effectiveness analyses suggest that PI implementation, like other measures for the improvement of blood safety, has an acceptable cost–benefit ratio in this specific application (34, 35). The potential cost savings from PI implementation could offset some costs associated with the technology (e.g., production costs); however, the amount of potential offsetting cost reductions may vary considerably between different countries and regions and must be evaluated on an individual basis for blood centers and hospitals (36). Finally, the available resources influence how politicians and health authorities decide on how to meet public concerns for safety in transfusion medicine. If emerging evidence continues to demonstrate the efficacy of PI, it will be difficult to explain to individuals with severe transfusion-associated infections why this readily available risk mitigation and safety measure was not implemented.

## Author Contributions

The author confirms being the sole contributor of this work and approved it for publication.

## Conflict of Interest Statement

The author works for German Red Cross Blood Service NSTOB, a blood donation center that collaborates with Macopharma on the development of a pathogen inactivation system for platelets. The author did not receive any financial support relevant to this manuscript.
